# Shrinkage of a pituitary metastasis of melanoma induced by pembrolizumab: a case report

**DOI:** 10.1186/s13256-021-03150-4

**Published:** 2021-11-11

**Authors:** Giuseppe Giuffrida, Francesco Ferraù, Ylenia Alessi, Salvatore Cannavò

**Affiliations:** 1 Endocrine Unit of University Hospital “G. Martino”, Messina, Italy; 2 Department of Human Pathology of Childhood and Adulthood “G. Barresi”, University of Messina, Messina, Sicily Italy

**Keywords:** Pituitary tumors, Pituitary metastases, Immune checkpoint inhibitors, Case report

## Abstract

**Background:**

Pituitary metastases are rare, often deriving from lung or breast cancer owing to the upper vena cava proximity. Pituitary metastases can manifest with signs and symptoms of pituitary tumors, consequent to mass effect (headache, visual impairment) and/or hormonal alterations (hyperprolactinemia, hypopituitarism, and diabetes insipidus). Immune checkpoint inhibitors burst immunity against tumors, significantly increasing patients’ survival, but their autoimmune side effects frequently involve the skin, the gastrointestinal tract, and the endocrine glands (pituitary, thyroid, pancreas).

**Case presentation:**

A 77-year-old Caucasian man had undergone trans-nasal sphenoidal surgery for a nonsecreting pituitary macroadenoma in 2001, without remnant or endocrine deficits. In 2016, he was operated for a shoulder melanoma. In February 2018, imaging evaluation demonstrated metastases in lung, liver, and femur. Therefore, treatment with pembrolizumab (anti-programmed death 1) was scheduled in May 2018, but, before starting this therapy, a brain computed tomography performed for a sudden loss of consciousness detected a sellar mass of 17 × 12 mm, which extended to the pituitary stalk and compressed the optic chiasma. Focused magnetic resonance imaging confirmed the size and characteristics of the lesion, while emergency evaluation of the hormonal profile demonstrated an impairment of adrenal and thyroid function. The pituitary lesion demonstrated a remarkable shrinkage (8 × 6 mm), which was confirmed by subsequent imaging evaluations.

**Conclusions:**

This is the first case reporting on effectiveness of immune checkpoint inhibitors in a patient with pituitary metastasis from a melanoma.

## Background

Pituitary metastases (PMs) are rare, accounting for 1% of all pituitary tumors and 0.4% of all intracranial metastatic tumors, respectively [1, 2]. They most frequently are a consequence of breast and lung cancer followed by renal and prostate carcinoma, with these four malignancies accounting for 71% of all PMs; nonetheless, cases from other solid tumors (melanoma/skin cancer, thyroid cancer, gastrointestinal carcinomas) as well as spread of leukemia, plasmacytomas, or multiple myelomas have been reported, with 3–4% of cases of unknown primary tumor [1, 3–5].

Clinically, PMs present with signs/symptoms in about 20% of patients only, occurring in end-stage disease [3]. The most frequent features are headache, visual field impairment, cranial neuropathy, and—concerning pituitary function—diabetes insipidus (DI) and hypopituitarism with one or more hormone deficits; apoplexy has been rarely reported, and sometimes in patients with preexisting functioning adenomas whose hypervascularization could favor the occurrence of metastases and apoplexy [2–4]. There are no specific radiological signs clearly indicating PMs, although in some series using magnetic resonance imaging (MRI) they tend to be isointense with brain parenchyma in both T1- and T2-weighted sequences, invading cavernous sinus or hypothalamus, and frequently present contrast enhancement. Moreover, sometimes these lesions display a dumbbell shape from a constriction at the diaphragma sellae level [3, 6, 7]. The loss of “bright spot” due to posterior infiltration, especially in patients with DI, is further frequent evidence [8].

Regarding PM treatment, surgery by trans-nasal sphenoidal (TNS) approach is often the first-line therapy, mainly considered for the relief of symptoms rather than for survival improvement that otherwise depends on the primary tumor [9]. Radiotherapy (RT) and radiosurgery are also available in these cases, while the role of chemotherapy is still confined to limited experiences [10, 11].

Immune checkpoint inhibitors (ICIs) are novel drugs targeting the molecular regulators of immune response to boost it against tumor tissue: anti-cytotoxic T-lymphocyte antigen 4 (CTLA4), programmed death 1 (PD-1), and its ligand (PD-1L) [12, 13]. So far, many papers in literature have demonstrated their efficacy at improving mean survival in cancer patients, but for the same reason ICIs are linked to a wide range of autoimmune reactions, mainly concerning the skin, the gastrointestinal tract, and the endocrine system [14, 15]. Endocrine adverse effects include hypophysitis, thyroid disorders (thyrotoxicosis or hypothyroidism), and type 1 diabetes mellitus (T1DM) [16].

Herein, we describe one case of PM that benefited greatly from treatment with an anti-PD1 (pembrolizumab) for advanced melanoma.

## Case presentation

A 77-year-old Caucasian male was referred to our endocrine unit in June 2018, for evaluation of a suspected PM from melanoma.

In 2001, the patient had been diagnosed with a nonsecreting pituitary macroadenoma of 25 mm in maximum diameter with suprasellar extension. He had been treated with TNS surgery in another center, and follow-up MRI studies did not demonstrate any residual tissue, up to 5 years after intervention, and pituitary function was preserved. Unfortunately, these images from the previous patient’s history are not available since he moved from another town.

In 2016, he had undergone surgical resection of a melanoma of the right shoulder, and then he was regularly followed up by an oncologist in another hospital, reporting no relapse after surgery.

**Fig. 1:**
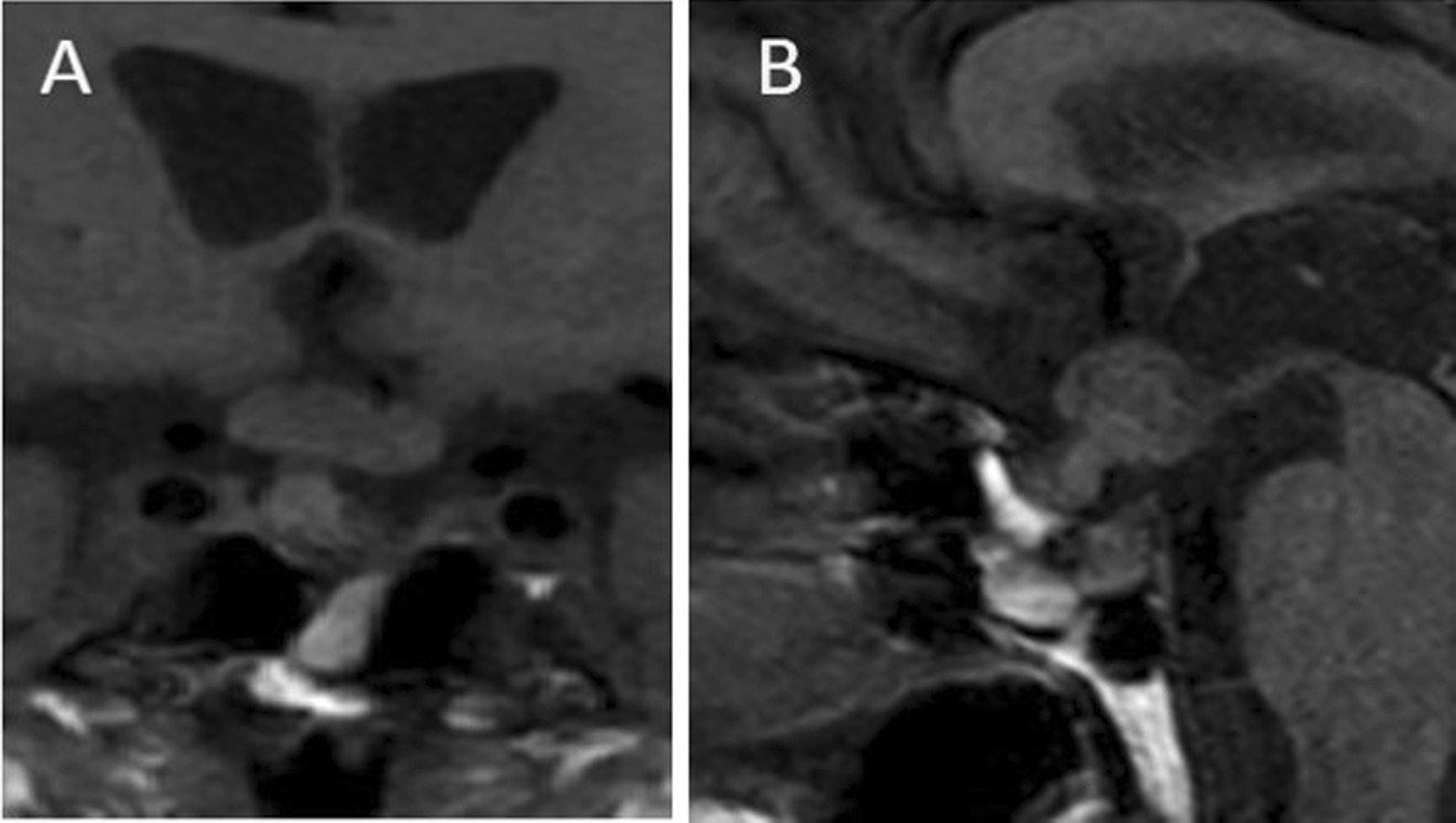
Focused sellar MRI study confirming the presence of a mass, isointense with the surrounding brain parenchyma in T1-weighted sequences and extending from the anterior pituitary towards the peduncle and the optical chiasma (the brain CT performed at emergency room (ER) admission demonstrated a sellar mass of about 17 × 12 mm), in coronal (**A**) and lateral (**B**) projection

In February 2018, however, imaging studies demonstrated secondary lesions at the lung, liver, and femur level; therefore, treatment with pembrolizumab (anti-PD1—*Keytruda*, MSD), 2 mg/kg in cycles of four intravenous infusions each with a 3-week interval, was scheduled. Nevertheless, at the beginning of May 2018 before starting this treatment, a sudden onset of nausea and dizziness followed by loss of consciousness required an emergency brain computed tomography (CT) evaluation, which revealed the appearance a sellar mass of about 17 × 12 mm. A focused sellar MRI confirmed the presence of this lesion, isointense with the surrounding parenchyma in T1-weighted sequences and extending from the anterior pituitary towards the peduncle and the optical chiasma (Fig. 1). For this reason, the patient underwent an emergency evaluation of adrenal and thyroid axes showing hormonal hypofunction and was prescribed with cortisone acetate (one and a half tablets per day) and levothyroxine (L-T4, 25 µg per day) by an endocrinology consultant. Then, he was discharged and referred to our endocrine unit for further investigations and adequate follow-up. At presentation, he did not report significant symptoms except diffuse weakness. He was also affected by arterial hypertension, appropriately controlled with nebivolol 5 mg per day, hypercholesterolemia treated with pravastatin 40 mg per day, osteoporosis managed by denosumab intramuscularly every 6 months and calcium carbonate 1000 mg per day, and stage II chronic kidney disease (CKD). He did not smoke and consumed only one glass of wine at meals. Physical examination demonstrated moderate overweight (weight 88 kg, height 180 cm, body mass index 27.2 kg/m^2^), normal heart rate at 72 beats per minute, and slightly high blood pressure (145/95 mmHg). Endocrine evaluation showed mild hyperprolactinemia (prolactin (PRL) 482 µIU/ml, normal range 86–324 µIU/ml) and panhypopituitarism, with an insufficient dose of thyroid hormone and an excess of urinary free cortisol (UFC) indicating corticosteroid overtreatment (Table 1). Furthermore, a visual field test was performed, demonstrating no impairment. So, replacement therapy with cortisone acetate and levothyroxine was adjusted as required, while testosterone and somatotroph hormone were not prescribed, according to patient’s preference and in consideration of the neoplastic progression, respectively. In the meantime, the patient had started pembrolizumab infusions since the end of May 2018.

**Table 1:** Patient’s biochemical profile at admission to our unit, after therapy adjustments and at the last follow-up visit

Parameters and units	Admission values (26 June 2018)	First follow-up visit (26 July 2018)	Last follow-up visit (24 June 2020)	Normal values
Prolactin (µIU/ml)	482	–	410.63	86–324
FSH (mIU/ml)	1.2	–	–	1.5–12.4
LH (mIU/ml)	0.3	–	–	1.7–8.6
Total testosterone (ng/dl)	2.50	–	–	193–386
IGF-1 (ng/ml)	36.34	–	–	47–141
Cortisol (µg/dl)	18.52	21.40	13.5	7–32
UFC (µg/24 hours)	428.06	274.60	–	30–350
FT4 (pmol/L)	10.30	15.60	12.61	12.0–22.0
TSH (µIU/ml)	0.033	0.023	0.02	0.27–4.2

Routine parameters were in the normal range (not reported), except for total cholesterol (236 mg/dl, normal range 130–220 mg/dl), while hormone assays demonstrated panhypopituitarism with hyperprolactinemia due to pituitary stalk compression. Replacement therapy with cortisone acetate (one and a half tablets per day) resulted in overdose, while levothyroxine (25 µg per day) was insufficient to reach normal FT4 levels (italics).

*FSH* follicle-stimulating hormone, *FT4* free thyroxine, *IGF-1* insulin-like growth factor 1, *LH* luteinizing hormone, *TSH* thyroid-stimulating hormone, *UFC* urinary free cortisol.

**Fig. 2:**
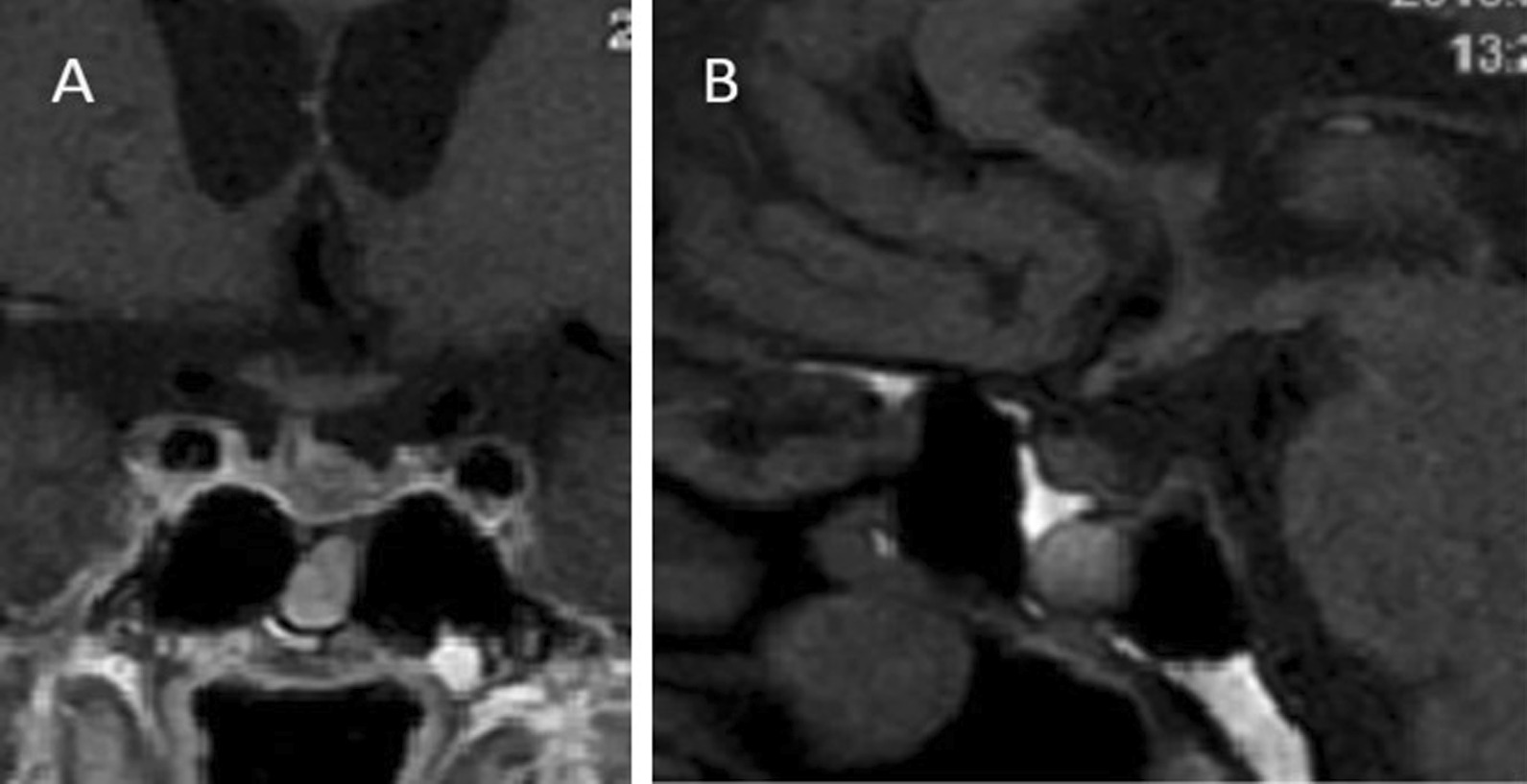
First post-treatment sellar MRI reevaluation in September 2018 demonstrating a significant volume decrease of the lesion, with a paramedian left 8 × 6 × 6 mm remnant, without alterations in the surrounding structures, in coronal (**A**) and lateral (**B**) projection

The whole-body positron-emission tomography (PET) and CT scans performed in August 2018 after one cycle of pembrolizumab infusions revealed reduced metabolic activity and morphovolumetric stability of the other known secondary lesions. The head-MRI study performed in September 2018 demonstrated a significant volume decrease (47%) of the pituitary lesion, thus confirming its secondary nature. In fact, only a paramedian left 8 × 6 × 6 mm remnant was described, without alterations in the surrounding structures (Fig. 2A, B). The same finding was confirmed at the following MRI controls in June 2019 and February 2020. Considering the patient’s conditions and his ongoing anticancer treatment, we did not try to withdraw any medication and test him for a possible hormone function improvement.

The patient is still followed up at our unit, while he continues his antineoplastic treatment under the oncologists’ supervision. His conditions remain good and stable under the current therapeutic regimen.

## Discussion and conclusions

Although rare, PMs should always be suspected in presence of a sudden worsening of symptoms, that is, indication of hypothyroidism (weakness, loss of memory, constipation), hypoadrenalism (hypotension, hypoglycemia, nausea, vomiting), sexual dysfunction (reduced libido, hair loss, erectile dysfunction), or headache or visual field impairment in a patient with neoplasia. DI is a frequent manifestation of PMs (about 50% of cases) since the posterior pituitary is directly perfused by the inferior hypophyseal arteries, lacking a portal system like the anterior lobe and thus being more susceptible to hematogenous spread. On the contrary, anterior pituitary deficiency is reported in 25–45% of cases, mainly manifesting itself with central hypothyroidism and secondary adrenal insufficiency [3, 5]. However, some reviews of the literature demonstrated a change in clinical presentation over recent years [17]. In fact, while symptomatic PMs presented with DI in 70% of cases and anterior dysfunction in 15% at the end of the 80s, the incidence of DI decreased to 32.8% versus 39.7% of hormone deficiency in 2015 [17]. Although the origin of this shift is unclear (publication bias, or different primary tumors and consequent predilection for metastases sites?), such evidence strengthens the importance of vague symptoms such as worsening hypotension, nausea, vomiting, etc. in cancer patients, especially with sudden onset [17].

There are few reports in the literature regarding pituitary metastases from melanoma. In a recent paper by Mattogno *et al.* reporting two cases from the authors’ practice, the lesions were characterized by similar histopathological features, appearing hemorrhagic with an Mib1 proliferation index of 15–20%, and one of them also presented with scattered deposits of melanin and BRAF^V600E^ mutation positivity [18]. PMs from melanoma are rare and usually occur at an advanced stage, with a mean age of onset around the fifties and no gender differences [18]. The tendency to bleed, with some cases of apoplexy being reported, and the melanotic pigmentation give these lesions a typical hyperintensity in T1-weighted MRI sequences [18, 19].

ICIs are successfully prolonging patients’ survival in the context of advanced solid tumors, but their use is often accompanied with autoimmune endocrine adverse events that are more frequent with the “oldest” anti-CTLA4 (that is, ipilimumab) than with the newest anti-PD1 and anti-PD1L (nivolumab, pembrolizumab, etc.), with the latter potentially causing more toxicity at the thyroid level [16]. The pathophysiology of such events is still partially unknown, but as observed in other experiences (that is, gastrointestinal toxicities), it could be related to an enhanced immune response from the activation of T cells by the monoclonal antibody, with a combined effect in the case of coupled administration (anti-CTLA4 *plus* anti-PD1/PD1L inhibitors) [20].

The case we present is paradigmatic for several reasons. First, the patient had already been treated for a pituitary macroadenoma almost a decade before, with neither evidence of remnant tissue nor hormone deficits for several years post-surgery. Second, the patient had demonstrated signs of pituitary dysfunction (hypoadrenalism with hypotension and syncope) before starting pembrolizumab, consistently with the mass effect exerted by the pituitary lesion. Third, pembrolizumab treatment dramatically led to pituitary mass shrinkage, confirming its secondary nature without any significant endocrine or metabolic side effect.

Some evidence of the efficacy exerted by novel anticancer treatments at the pituitary/central nervous system level is emerging in the literature, such as a recent report describing the responsiveness to ICIs by a pituitary adrenocorticotropic hormone (ACTH)-secreting carcinoma [21]. In this case, a 35-year-old woman was treated with a combination of ipilimumab (3 mg every 3 weeks) and nivolumab (3 mg every 3 weeks), with a dramatic biochemical response and shrinkage of the pituitary and liver lesions. It should be considered that this patient also received six cycles of temozolomide (TMZ)/capecitabine that could have induced a hypermutation in the neoplastic cells, thus favoring the positive response to ICI treatment [21, 22]. Moreover, some studies have demonstrated the expression of PD-1 in benign pituitary adenomas, regardless of other prognostic factors like recurrence, proliferative index, or aggressiveness, and it seems that its levels were higher in secreting versus nonfunctioning adenomas [23, 24].

PMs can be included in the broader category of brain metastases (BMs), which complicate neoplastic diseases in 8–10% of cases, with the highest cumulative incidence in melanoma [25]. In these cases, the role of systemic therapies is limited due to the difficulty of penetrating the brain through the blood–brain barrier (BBB), differently from ICIs that modulate T-cell activity [26]. Four studies on pembrolizumab monotherapy in melanoma BMs reported an intracranial objective response rate (ORR) of 22–40%, and an overall survival (OS) of 17–20.4 months, with a median progression-free survival (PFS) interval of 2–5.2 months [27]. Median OS and PFS reached 17.4 and 2.9 months, respectively, with the combination of CTLA4 inhibitors and anti-PD1 (that is, ipilimumab *plus* pembrolizumab) [27, 28]. The cumulative median OS in these studies was only 8 months but was slightly higher for melanoma than for small-cell lung carcinoma (SCLC) BMs, with a possible link to the tumor microenvironment [28]. In fact, a high concentration of tumor infiltrating lymphocytes (TILs), often present in secondary lesions deriving from melanoma, has been associated with improved survival [29]. Besides, the administration of corticosteroids to reduce peritumoral brain edema could impair ICI efficacy by leading to an immunosuppression state [30]. An emergent therapeutic approach is the combination of ICIs with RT, whose promising role could be linked to the abscopal effect, that is, the modifications in cancer cell DNA and cytokine/chemokine secretion producing a better interaction with the immune system and its modulators, with both local and systemic improvement [29]. A recent meta-analysis investigated the combination ICI-hypofractionated radiotherapy (HFRT, 5–20 Gy per fraction), demonstrating a significant increase in 6-month locoregional recurrence-free survival (LRFS), 6-month OS rate, 1-year OS rate, 2-year OS rate, and median OS [31]. Of note, no significant increase in toxicities was observed in comparison with HFRT alone or HFRT plus chemotherapy or other systemic therapies [31]. However, the exact timing of combination, the optimal radiation dose or fractionation protocol, and other points should be clarified by means of large clinical trials. Finally, as reported in recent papers, in the case of PMs deriving from melanoma with mutated BRAF^V600E^, an intriguing therapeutical perspective is represented by BRAF inhibitors (that is, dabrafenib combined with trametinib, a mitogen-activated extracellular signal-regulated kinase inhibitor) that could add another pharmacological approach to these lesions [18].

In conclusion, this clinical case reminds us of the possibility of PMs originating not only from breast or lung cancer as in most cases, but also from other tumors like melanoma, thus highlighting the importance of accurate follow-up of cancer patients to identify potential pituitary involvement. It generally occurs at an advanced stage, and the onset is often sudden and dramatic, due to hormonal dysfunction and/or mass effect. Finally, this report, for the first time, sheds light on the role of ICIs in the management of PMs from “ICI-sensitive” malignancies, considering that often surgery is not feasible in such patients. In these cases, targeting the immune checkpoints could significantly prolong the OS rate. Larger studies comparing the role of ICIs, alone or combined with each other or with RT in PMs from “ICI-sensitive” cancers, should be carried on to extend their use in these rare and complicated clinical settings.

## Data Availability

All data generated or analysed during this study are included in this published article.

## Abbreviations

PMs: Pituitary metastases
ICIs: Immune checkpoint inhibitors
TNS: Trans-nasal sphenoidal
CT: Computed tomography
MRI: Magnetic resonance imaging
DI: Diabetes insipidus
RT: Radiotherapy
CTLA4: Cytotoxic T-lymphocyte antigen 4
PD-1: Programmed death 1
PD-1L: Programmed death 1 ligand
T1DM: Type 1 diabetes mellitus
CKD: Chronic kidney disease
PET: Positron-emission tomography
AITDs: Autoimmune thyroid diseases
ORR: Objective response rate
TMZ: Temozolomide
PFS: Progression-free survival
BMs: Brain metastases
BBB: Blood–brain barrier
SCLC: Small-cell lung carcinoma
HFRT: Hypofractionated radiotherapy
LRFS: Locoregional recurrence-free survival
